# G-CSF + plerixafor versus G-CSF alone mobilized hematopoietic stem cells in patients with multiple myeloma and lymphoma: a systematic review and meta-analysis

**DOI:** 10.1080/07853890.2024.2329140

**Published:** 2024-03-12

**Authors:** Yuyao Li, Xia Qiu, Yupeng Lei, Ruixi Zhou

**Affiliations:** a Key Laboratory of Birth Defects and Related Diseases of Women and Children, Sichuan University, Ministry of Education, Chengdu, Sichuan, China; b Department of Obstetrics and Gynecology, West China Second University Hospital, Sichuan University, Chengdu, Sichuan, China; c Department of Pediatrics, West China Second University Hospital, Sichuan University, Chengdu, Sichuan, China

**Keywords:** Plerixafor, G-CSF, CD34+ cell, First-line, MM, NHL, HL

## Abstract

**Aim:**

The combination of granulocyte-colony stimulating factor (G-CSF) and plerixafor is one of the approaches for hematopoietic stem cell mobilization in patients with multiple myeloma (MM), non-Hodgkin’s lymphoma (NHL), and Hodgkin’s lymphoma (HL). This systematic review and meta-analysis aimed to determine the ability of G-CSF + plerixafor to mobilize peripheral blood (PB) CD34+ cells and examine its safety profile.

**Methods:**

We performed a database search using the terms ‘granulocyte colony stimulating factor’, ‘G-CSF’, ‘AMD3100’, and ‘plerixafor’, published up to May 1, 2023. The methodology is described in further detail in the PROSPERO database (CRD42023425760).

**Results:**

Twenty-three studies were included in this systematic review and meta-analysis. G-CSF + plerixafor resulted in more patients achieving the predetermined apheresis yield of CD34+ cells than G-CSF alone (OR, 5.33; 95%, 4.34–6.55). It was further discovered that G-CSF + plerixafor could mobilize more CD34+ cells into PB, which was beneficial for the next transplantation in both randomized controlled (MD, 18.30; 95%, 8.74–27.85) and single-arm (MD, 20.67; 95%, 14.34–27.00) trials. Furthermore, G-CSF + plerixafor did not cause more treatment emergent adverse events than G-CSF alone (OR, 1.25; 95%, 0.87–1.80).

**Conclusions:**

This study suggests that the combination of G-CSF and plerixafor, resulted in more patients with MM, NHL, and HL, achieving the predetermined apheresis yield of CD34+ cells, which is related to the more effective mobilization of CD34+ cells into PB.

## 1. Introduction

Multiple myeloma (MM), non-Hodgkin’s lymphoma (NHL), and Hodgkin’s lymphoma (HL) are common hematological malignancies, which often lead to complications and a decreased quality of life for patients [1–3]. To treat the myelosuppression caused by these disorders, autologous hematopoietic stem cell transplantation (AHSCT) is recommended to restore bone marrow and hematopoietic functions after high-dose chemotherapy, as well as to generate immunity [4–6]. Compared to conventional chemotherapy, AHSCT improves patient remission and overall survival [7, 8]. Clinically, isolating sufficient CD34+ cells from peripheral blood (PB) for transplantation is necessary, among which hematopoietic stem cells (HSCs) account for more than 90% of the CD34+ cells [9].

In clinical practice, PB CD34+ cells mobilized by Granulocyte colony-stimulating factor (G-CSF) are often used for AHSCT, revolutionizing stem cell transplantation [10, 11]. Different forms of recombinant human G-CSF, such as filgrastim, lenograstim, and pegfilgrastim, are widely used in clinical practice, reducing febrile neutropenia and other adverse reactions [12]. After the patent period expired, some biosimilars have been developed, but their efficacy and safety still need to be further verified [10]. However, the administration of G-CSF alone may lead to insufficient collection of CD34+ cells, causing mobilization failure. The risk factors for mobilization failure include age of the patient, the presence of T-cell and NK/T-cell lymphoma, and high-dose cyclophosphamide treatment [13]. In recent years, plerixafor, a CXCR4 receptor-specific inhibitor, has been proposed for administration in combination with G-CSF for HSC mobilization, and that this combination resulted in a higher collection of CD34+ cells in patients with MM, NHL, and HL compared to G-CSF alone [14–16]. Studies have shown that G-CSF + plerixafor cannot achieve the desired therapeutic effect in patients who have already undergone mobilization without success using G-CSF alone [17–19]. Therefore, whether G-CSF + plerixafor has clinical value for mobilizing CD34+ cells in patients with MM, NHL, and HL and for patients with mobilization failure warrants further investigation.

Accordingly, we conducted a systematic review and meta-analysis to evaluate the clinical efficacy and safety of G-CSF + plerixafor for CD34+ cell mobilization in patients with MM, NHL, and HL.

## 2. Methods

### 2.1. Search strategy and selection criteria

The study protocol followed the Preferred Reporting Items for Systematic Reviews and Meta-Analyses-Diagnostic Test Accuracy (PRISMA-DTA) statement and was registered in the PROSPERO database (ID: CRD42023425760) in May 2023. We searched the database using the following term: ‘plerixafor’, ‘AMD3100’, ‘G-CSF’, and ‘granulocyte colony-stimulating factor’. PubMed, Embase, Cochrane, and Web of Science databases were searched for potentially relevant studies in English-language articles (up to May 1, 2023). References to the identified articles were also searched to supplement the data sources.

### 2.2. Study selection

Titles and abstracts were independently screened by two reviewers after removing duplicate studies retrieved from the four databases and other sources [20]. Disagreements were resolved through consultation, and third-party opinions were consulted when necessary. Studies that met the following criteria were included in the meta-analysis: (1) Full-text studies published in peer-reviewed journals. (2) Patients who were diagnosed with MM, NHL, or HL; and (3) received G-CSF + plerixafor to mobilize CD34+ cells. (4) Patients who also had at least one of the following indicators: those who achieved the predetermined apheresis CD34+ cell yield (cells × 10^6^/kg) and those with available CD34+ cell counts (cells/μL). The exclusion criteria were as follows: (1) reviews, abstracts, comments, and case reports; (2) non-English language publications; and (3) studies consisting of less than five patients.

### 2.3. Risk of bias assessment

Two reviewers independently assessed the risk of bias in eligible studies using the Cochrane ‘risk-of-bias’ assessment tool.

### 2.4. Data extraction and statistical analysis

Two reviewers independently extracted data from eligible studies, including the following: author, year, trial ID, patients (age and disease), administration (G-CSF and plerixafor), predetermined apheresis yield of CD34+ cells, confounding variables, and previous treatment of the patients. Data analysis was performed using the RevMan software. A meta-analysis was carried out on the data about the administration of ‘G-CSF + plerixafor’ versus ‘G-CSF alone’ or before and after the addition of plerixafor: (1) number of patients who achieved the predetermined apheresis yield of CD34+ cell count (cells × 10^6^/kg), (2) CD34+ cell count (cells/μL), and (3) number of adverse events. Subgroup analysis was performed according to the disease type. An I^2^ > 50% was considered inappropriate heterogeneity, in which case the random-effects model was used. Statistical significance was set at *p* < 0.05.

## 3. Results

### 3.1. Search results

**Figure 1.**
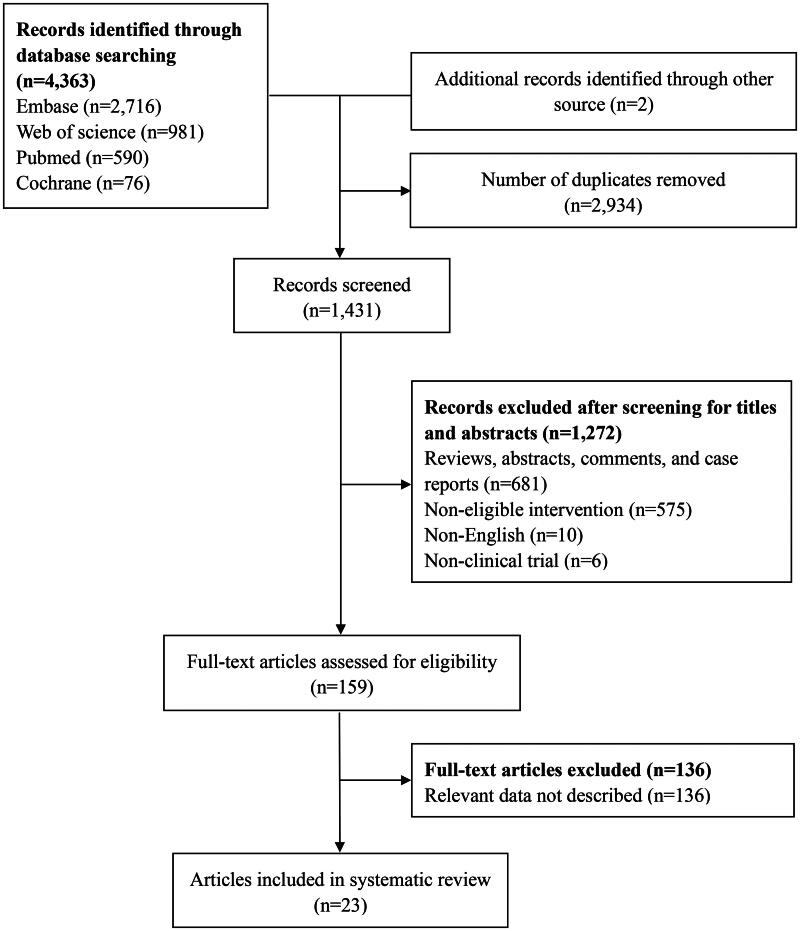
Search strategy and study selection.

A total of 4,363 records were retrieved from the four databases according to the search terms, and two records were retrieved from other sources. Duplicate records were excluded, and a total of 23 articles were finally included in this systematic review and meta-analysis after applying the inclusion and exclusion criteria (Figure 1) [17–19, 21–40].

### 3.2. Studies characterisations

Table 1 lists the main characteristics of the 23 included articles. The articles were published between 2008 and 2023, were single- or multicenter, prospective, or retrospective studies, and were listed with clinical trial IDs. All patients were adults with MM, NHL, or HL. The dose of G-CSF was mainly 10 μg/kg, and the dose of plerixafor was mainly 0.24 mg/kg. The predetermined apheresis yield of CD34+ cells was between 2 × 10^6^ and 6 × 10^6^ cells/kg. In addition, Supplementary Table S1 lists the confounding variables and previous treatment of the patients.

**Table 1.** Main characteristics of included studies.

Author	Year	Study type	Trial ID	Patient		Administration		Predtermined apheresis yield of CD34+ cell count (cells/kg)
				Age	Disease	G-CSF	Plerixafor	
Steiner	2023	Single-center, retrospective	NA	Adult	NHL, HL, and MM	7.5 μg/kg	24 mg as a fixed dose	2 × 10^6^ for NHL and HL 4 × 10^6^ for MM
Morris	2020	Multi-center, prospective	NCT01362972	Adult	MM	NA	NA	4 × 10^6^
Ray	2020	Single-center, prospective	NA	Adult	MM	10 μg/kg	0.24 mg/kg (Crcl higher than 50 mL/min) 0.16 mg/kg (Crcl was 30-49 mL/min)	5 × 10^6^
Sureda	2020	Multi-center, prospective	NCT01362972	Adult	NHL and HL	NA	NA	1 × 10^6^
Nahi	2019	Multi-center, prospective	NCT01753453	Adult	MM	0.24 μg/kg	0.24 mg/kg	2 × 10^6^
Matsue	2018	Single-center, prospective	NCT02221492	Adult	NHL	400 μg/m^2^	0.24 mg/kg	5 × 10^6^
Zhu	2018	Multi-center, prospective	NCT01767714	Adult	NHL	10 μg/kg	0.24 mg/kg	5 × 10^6^
Haverkos	2017	Single-center, prospective	NA	Adult	NHL and HL	10 μg/kg	0.24 mg/kg	2 × 10^6^
Ri	2017	Single-center, prospective	NCT02221479	Adult	MM	400 μg/m^2^	0.24 mg/kg	6 × 10^6^
Sánchez-Ortega	2015	Multi-center, retrospective	NA	Adult	NHL, HL, and MM	NA	NA	2 × 10^6^
Maziarz	2013	Single-center, retrospective	NA	Adult	NHL	10 μg/kg	0.24 mg/kg	5 × 10^6^
Micallef	2013	Retrospective	NA	Adult	NHL and MM	10 μg/kg	0.24 mg/kg	5 × 10^6^ for NHL 6 × 10^6^ for MM
Andreola	2012	Prospective	NA	Adult	NHL, HL, and MM	10 μg/kg	0.24 mg/kg	2 × 10^6^ for NHL and HL 4 × 10^6^ for MM
Varmavuo	2012	Single-center, prospective	NA	Adult	NHL	5 μg/kg (filgrastim) or 6/12 mg as a fixed dose (pegfilgrastim)	12-24 mg as a fixed dose	2 × 10^6^
Cooper	2011	Single-center, retrospective	NA	Adult	NHL and MM	10 μg/kg or 16 μg/kg twice for poor mobilization	0.24 mg/kg	5 × 10^6^
Horwitz	2011	Single-center, prospective	NA	Adult	NHL, HL, and MM	10 μg/kg	0.24 mg/kg	2 × 10^6^
Worel	2011	Multi-center, prospective	NA	Adult	NHL and MM	10 μg/kg	0.24 mg/kg	2 × 10^6^ for NHL 4 × 10^6^ for MM
DiPersio a	2009	Multi-center, prospective	NCT00103662	Adult	MM	10 μg/kg	0.24 mg/kg	6 × 10^6^
DiPersio b	2009	Multi-center, prospective	NA	Adult	NHL	10 μg/kg	0.24 mg/kg	5 × 10^6^
Stewart	2009	Multi-center, prospective	NCT00396266	Adult	NHLand MM	10 μg/kg	0.24 mg/kg	5 × 10^6^
Stiff	2009	Single-center, prospective	NCT00322491	Adult	NHLand MM	10 μg/kg	0.24 mg/kg	5 × 10^6^
Cashen	2008	Single-center, prospective	NA	Adult	HL	10 μg/kg	0.24 mg/kg	5 × 10^6^
Pusic	2008	Single-center, retrospective	NA	Adult	NHL, HL, and MM	10 μg/kg	0.24 mg/kg	2 × 10^6^

G-CSF, granulocyte colony-stimulating factor; HL, Hodgkin’s lymphoma; MM, multiple myeloma; NA, not available; NHL, non-Hodgkin’s lymphoma.

### 3.3. Risk of bias in studies

**Figure 2.**
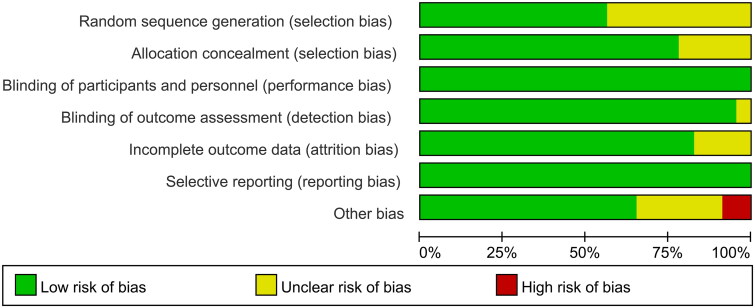
Risk of bias graph in the included studies.

We assessed the risk of bias in the included articles (Figure 2), and the results are summarized in Supplementary Figure S1. ‘Blinding of patients and personnel’ and ‘selective reporting’ of eight articles [17–19, 21–39] were assessed as low risk. In ‘random sequence generation’, bias in ten articles [17–19, 23, 24, 28, 35, 38–40] was assessed as unclear risk. In ‘allocation concealment’, bias in five articles [17–19, 28, 35, 38] was assessed as unclear risk. In ‘blinding of outcome assessment’, bias in one article [33] was assessed as unclear risk. In ‘incomplete outcome data’, bias in four articles [17, 19, 30, 39] was assessed as unclear risk. Among ‘other biases’, bias in two articles [31, 40] was assessed as high risk, and bias in six articles [17, 18, 26, 29, 32, 39] was assessed as unclear risk. In general, the risk of bias was low.

### 3.4. G-CSF + plerixafor enabled more patients to achieve the predetermined apheresis yield of CD34+ cells

**Figure 3.**
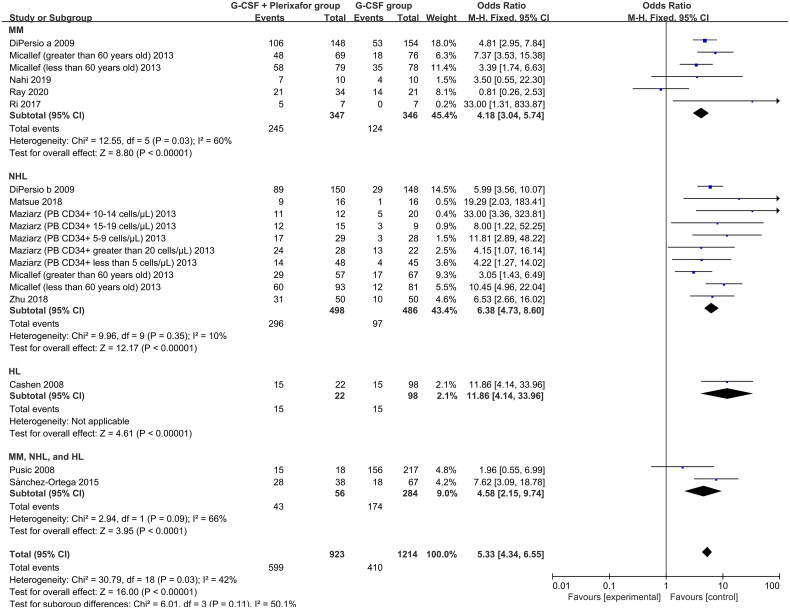
Meta-analysis of the proportion of patients achieving the predetermined apheresis yield of CD34+ cells (cells × 10^6^/kg) in the G-CSF + plerixafor group versus the G-CSF group. HL, Hodgkin’s lymphoma; MM, multiple myeloma; NHL, non-Hodgkin’s lymphoma; PB, peripheral blood.

For patients requiring AHSCT, the clinical efficacy of G-CSF + plerixafor was evaluated based on the proportion of patients who achieved the predetermined apheresis yield of CD34+ cells. In twelve articles, 923 patients in the G-CSF + plerixafor group and 1214 patients in the G-CSF group were included in the meta-analysis [22, 26–33, 36, 37, 40]. Overall, more patients in the G-CSF + plerixafor group (64.9%, 599/923) achieved the predetermined apheresis yield of CD34+ cells compared to G-CSF alone (33.8%, 410/1214) (OR, 5.33; 95% CI, 4.34–6.55; *p* < 0.00001) (Figure 3). Subgroup analysis demonstrated significantly higher odds ratios for achieving the predetermined apheresis yield of CD34+ cells in the MM (OR, 4.18; 95% CI, 3.04–5.74; *p* < 0.00001), NHL (OR, 6.38; 95% CI, 4.73–8.60; *p* < 0.00001), HL (OR, 11.86; 95% CI, 4.14–33.96; *p* < 0.00001), and (MM, NHL, and HL) groups (OR, 4.58; 95% CI, 2.15–9.74; *p* < 0.0001) when administering G-CSF + plerixafor compared to G-CSF alone. The above results suggest that the addition of plerixafor could enhance the activity of G-CSF to isolate more CD34+ cells, which is conducive to the successful AHSCT of patients.

### 3.5. G-CSF + plerixafor was not effective in increasing CD34+ cells in patients who had failed to mobilize with G-CSF alone

Owing to its high price and other factors, plerixafor is not frequently used as a primary therapy, but is often used as a salvage therapy (also known as ‘just in time’) after mobilization failure using G-CSF alone [41]. In five articles, 396 patients in the G-CSF + plerixafor group and 328 patients in the G-CSF group were included in the meta-analysis [17–19, 22, 35, 41]. Overall, even in patients whose mobilization failed using G-CSF alone (89.9%, 295/328), salvage addition of plerixafor (57.1%, 226/396) resulted in a lower population ratio of achieved predetermined apheresis CD34+ cells than in patients who successfully mobilized with G-CSF alone (OR, 0.14; 95% CI, 0.06–0.29; *p* < 0.00001) (Supplementary Figure S2). Subgroup analysis showed that in both the MM (OR, 0.16; 95% CI, 0.04–0.68; *p* = 0.001) and (NHL and HL) groups (OR, 0.09; 95% CI, 0.03–0.29; *p* < 0.0001), the number of people who achieved the predetermined apheresis yield of CD34+ cells was still lower after adding plerixafor to the mobilization of those who failed compared to the mobilization of those who succeeded with G-CSF alone. In the NHL group, adding plerixafor could improve mobilization (OR, 0.29; 95% CI, 0.08–1.13; *p* = 0.07); however, only one study investigated this. These results suggest that the salvage addition of plerixafor does not contribute to achieving the predetermined apheresis CD34+ cell count in patients who had already failed to mobilize with G-CSF alone.

### 3.6. G-CSF + plerixafor increased the PB CD34+ cell count

**Figure 4.**
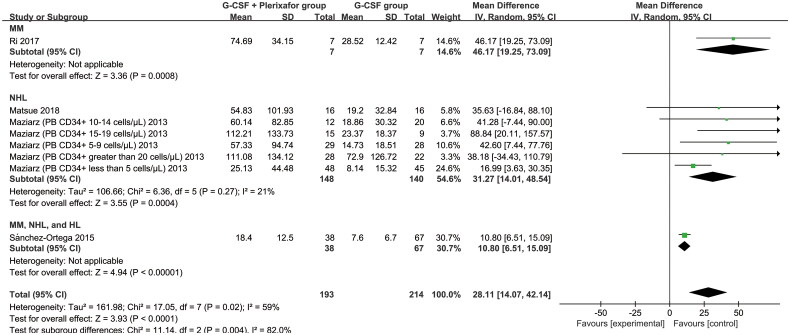
Meta-analysis of PB CD34+ cell count (cells/μL) between the G-CSF + plerixafor and G-CSF groups. HL, Hodgkin’s lymphoma; MM, multiple myeloma; NHL, non-Hodgkin’s lymphoma; PB, peripheral blood.

We further investigated the enhancing effect of plerixafor on the proliferation of PB CD34+ cells regardless of patient mobilization failure. In four articles, 193 patients in the G-CSF + plerixafor group and 214 patients in the G-CSF group were included in the meta-analysis [26, 27, 32, 33]. Overall, we found that the PB CD34+ cell count was significantly higher in the G-CSF + plerixafor group than in the G-CSF group (MD, 28.11; 95% CI, 14.07–42.14; *p* = 0.004) (Figure 4). Subgroup analysis showed that PB CD34+ cells in the MM (MD, 46.17; 95% CI, 19.25–73.09; *p* = 0.0008), NHL (MD, 31.27; 95% CI, 14.01–48.54; *p* = 0.0004), and (MM, NHL, and HL) groups (MD, 10.80; 95% CI, 6.51–15.09; *p* < 0.00001) were higher with G-CSF + plerixafor than with G-CSF alone; however, no difference was observed in the MM group (MD, 22.30; 95% CI, −20.09–64.69; *p* = 0.30). These results suggest that, compared to G-CSF alone, G-CSF + plerixafor could improve the PB CD34+ cell count.

### 3.7. PB CD34+ cell count was higher after G-CSF combined with plerixafor

**Figure 5.**
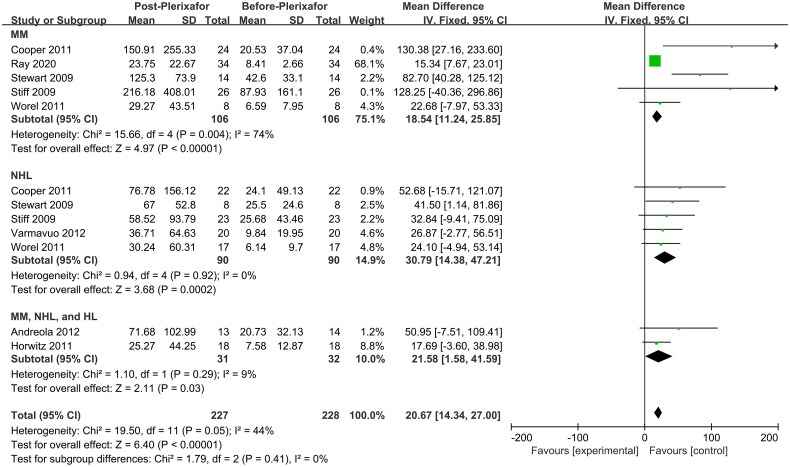
Meta-analysis of PB CD34+ cell count (cells/μL) before and after plerixafor administration. PB, peripheral blood. HL, Hodgkin’s lymphoma; MM, multiple myeloma; NHL, non-Hodgkin’s lymphoma.

To effectively compare the changes in PB CD34+ cell counts before and after the addition of plerixafor, 228 patients (one withdrawn) from eight single-arm studies were included [22–25, 28, 34, 38, 39]. In general, the PB CD34+ cell count was significantly higher after G-CSF treatment combined with plerixafor compared to the initial count (MD, 20.67; 95% CI, 14.34–27.00; *p* < 0.00001) (Figure 5). Similar results were obtained for the MM (MD, 18.54; 95% CI, 11.24–25.85; *p* < 0.00001), NHL (MD, 30.79; 95% CI, 14.38–47.21; *p* = 0.0002), and (MM, NHL, and HL) groups (MD, 21.58; 95% CI, 1.58–41.59; *p* = 0.03) in subgroup analyses. These results suggest that G-CSF combined with plerixafor increased the PB CD34+ cell count.

### 3.8. G-CSF combined with plerixafor did not increase treatment emergent adverse events (TEAEs)

**Figure 6.**
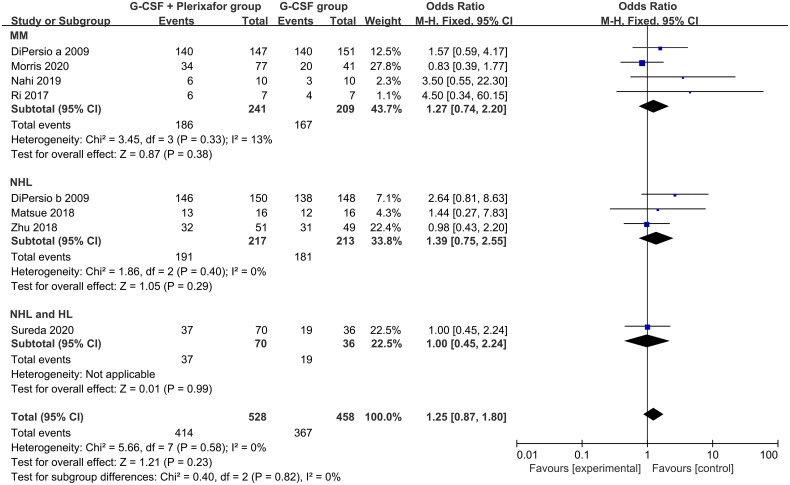
Meta-analysis of the incidence of TEAEs between the G-CSF + plerixafor group versus the G-CSF group. HL, Hodgkin’s lymphoma; MM, multiple myeloma; NHL, non-Hodgkin’s lymphoma; TEAEs, treatment emergent adverse events.

The evaluation of the safety of plerixafor mainly depends on treatment emergent adverse events (TEAEs). In eight articles, 528 patients in the G-CSF + plerixafor group and 458 patients in the G-CSF group were included in the meta-analysis [17, 19, 21, 27, 30, 32, 36, 37]. In general, G-CSF + plerixafor (78.4%, 414/528) did not cause more TEAEs than G-CSF alone (80.1%, 367/458) (OR, 1.25; 95% CI, 0.87–1.80; *p* = 0.23) (Figure 6). Subgroup analysis found that the addition of plerixafor in the MM (OR, 1.27; 95% CI, 0.74–2.20; *p* = 0.38), NHL (OR, 1.39; 95% CI, 0.75–2.55; *p* = 0.29), and (NHL and HL) groups (OR, 1.00; 95% CI, 0.45–2.24; *p* = 0.99) did not affect the incidence of TEAEs. These results suggest that plerixafor did not cause more TEAEs during the mobilization of CD34+ cells using G-CSF.

## 4. Discussion

This systematic review and meta-analysis found that G-CSF administered in combination with plerixafor mobilized CD34+ cells more effectively than G-CSF alone in patients with MM, NHL, and HL. G-CSF + plerixafor increased the number of patients reaching the predetermined apheresis yield of CD34+ cells, increased the PB CD34+ cell count, and did not cause additional TEAEs, thus increasing the success rate of AHSCT. In addition, timely plerixafor supplementation also helped to increase PB CD34+ cell count in patients who failed mobilization with G-CSF alone.

For patients with malignant hematological diseases, such as MM, NHL, and HL, AHSCT can potentially prolong survival [42–44]. Whether sufficient HSCs can be mobilized is the key to the success of transplantation. G-CSF is a commonly used pre-transplant to mobilize HSCs because it is safe and produces rapid and sustained engraftment [45–47]. However, some patients cannot be effectively mobilized using G-CSF alone. Recently, plerixafor has been recommended for administration in combination with G-CSF, which has been shown to mobilize HSCs to the PB more efficiently [48, 49]. However, due to its high price, plerixafor is rarely used in combination with G-CSF during the first mobilization [50]. Therefore, evaluating whether the combination of plerixafor and G-CSF is more capable of mobilizing HSCs is necessary, along with determining whether it is safe.

The combination of G-CSF and plerixafor demonstrated a higher proportion of patients achieving the predetermined apheresis yield of CD34+ cells compared to G-CSF alone, regardless of whether the patients were refractory, relapsed, or failed to mobilize (Figure 3). This directly benefits the subsequent AHSCT. In addition, that the predetermined apheresis yield of CD34+ cells was lower in patients with NHL and HL compared to patients with MM was noteworthy, which may be due to the fact that patients with NHL and HL tend to have longer treatment lines, which may affect the effectiveness of mobilization. However, for poor mobilizers using G-CSF alone, isolating the predetermined apheresis yield of CD34+ cells was difficult, even after salvage addition of plerixafor (Supplementary Figure S2). These results suggest that the timely administration of plerixafor can better mobilize CD34+ cells than salvage administration. The failure of salvage administration may be attributed to medical policy. Due to the need to limit medical expenses, plerixafor is often administered to the poorest mobilizers at the highest risk [51]. Many patients with significant mobilization deficits do not achieve the predetermined apheresis yield of CD34+ cells after receiving salvage plerixafor [17–19, 23, 35]. Notably, the bias variable was that different original studies used different predetermined apheresis CD34+ cell yields. In general, 2 × 10^6^ CD34+ cells/kg is considered the minimum acceptable cell yield, and 4 × 10^6^ CD34+ cells/kg or higher is considered optimal [52]. The predetermined apheresis CD34+ cell yields for MM is generally higher than those for NHL or HL [18, 22, 31, 39].

G-CSF promotes the release of CD34+ cells from the bone marrow niche to the PB by interrupting signaling pathways involved in motility such as CXCL12/CXCR4 [53]. Plerixafor further mobilizes CD34+ cells into the PB, which is conducive for collection [52]. In our study, compared to the G-CSF only group, PB CD34+ cells were increased in the G-CSF + plerixafor group (Figure 4). After G-CSF treatment alone failed to collect enough PB CD34+ cells, timely plerixafor supplementation contributed to a subsequent increase in PB CD34+ cell numbers (Figure 5). However, determining the optimal timing of plerixafor supplementation and the mode of administration require further clinical research. Moreover, we found that PB CD34+ cells mobilized in patients with MM were consistently lower than those in patients with NHL regardless of the mobilization protocol, which may be related to the high cytogenetic or molecular genetic risk present in patients with MM [54]. We confirmed from multiple sources that the combination of G-CSF and plerixafor could mobilize PB CD34+ cells better.

The combination of G-CSF and plerixafor was also promising in terms of safety, as it caused no additional TEAEs than G-CSF alone (Figure 6). Overall, the grades of plerixafor-associated TEAEs were low; no grade 4 or 5 TEAEs were reported. In addition, no deaths were reported. Grades 1 and 2 TEAEs included gastrointestinal disorders and injection site reactions, whereas grade 3 TEAEs included peripheral neuropathy and pleural effusion. In addition, that plerixafor did not cause poor engraftment of HSCs, but rather improved bone marrow regeneration was gratifying. Results indicated that the combined mobilization due to G-CSF and plerixafor resulted in rapid and durable engraftment of HSCs.

However, our systematic review and meta-analysis study still has some limitations. One limitation is that NHL includes a variety of disease entities. However, due to the difficulty in extracting data from the original studies, making a more detailed classification was not possible, which limited the precision of our study.

## 5. Conclusions

This study found that the combination of G-CSF and plerixafor was more likely to achieve a predetermined apheresis yield of CD34+ cells than G-CSF alone in patients with MM, NHL, and HL. This is mainly related to the fact that plerixafor can mobilize HSCs to the PB more effectively. Early combined administration of plerixafor and G-CSF may be helpful for mobilization, but it is of limited help to poor mobilizers who already received G-CSF alone. Moreover, the combination of G-CSF and plerixafor did not cause severe TEAEs. Therefore, the combination of G-CSF and plerixafor is beneficial to AHSCT.

## Supplementary Material

Supplemental Material

## Data Availability

Data are available to investigators after request.

## Abbreviations

G-CSF: Granulocyte colony-stimulating factor
HSC: Hematopoietic stem cells
MM: Multiple myeloma
NHL: Non-Hodgkin’s lymphoma
HL: Hodgkin’s lymphoma
PB: Peripheral blood
AHSCT: Autologous hematopoietic stem cell transplantation
TEAE: Treatment emergent adverse events
