# Supplementary material for: G-CSF + plerixafor versus G-CSF alone mobilized hematopoietic stem cells in patients with multiple myeloma and lymphoma: a systematic review and meta-analysis
Source: Ann Med. 2024 Mar 12;56(1):2329140. doi: 10.1080/07853890.2024.2329140 (PMC10939106; doi:10.1080/07853890.2024.2329140)
Supplement: Supplemental Material [file IANN_A_2329140_SM1114.zip › Supplementary Figures.doc]

**Supplementary figure 1.** Risk of bias summary in included studies.

**Supplementary figure 2.** Meta-analysis of the proportion of patients achieving the ideal yield of CD34+ cell count in the G-CSF + plerixafor group (as salvage therapy) versus the G-CSF group. HL, Hodgkin’s lymphoma; MM, multiple myeloma; NHL, non-Hodgkin’s lymphoma.
