# Supplementary material for: G-CSF + plerixafor versus G-CSF alone mobilized hematopoietic stem cells in patients with multiple myeloma and lymphoma: a systematic review and meta-analysis
Source: Ann Med. 2024 Mar 12;56(1):2329140. doi: 10.1080/07853890.2024.2329140 (PMC10939106; doi:10.1080/07853890.2024.2329140)
Supplement: Supplemental Material [file IANN_A_2329140_SM1114.zip › Supplementary Table 1.doc]

**Supplementary Table 1.** Confounding variables and previous treatment of the patients.

| Author | Year | Confounding variables | Previous treatments |  |
| --- | --- | --- | --- | --- |
|  |  |  |  |  |
| Steiner | 2023 | Did not proceed to stem cell harvest (n = 3);  Did not proceed to transplant either because of patient refusal, transplant ineligibility, or for other reasons (n = 30) | Patients with Multiple Myeloma: Plerixafor + G-CSF Group: 100% had prior therapy once, 36% had prior radiotherapy;  G-CSF alone Group: 94% had prior therapy once, 3% had prior therapy twice, 3% had prior therapy more than 3 times, 27% had prior radiotherapy.  Patients with Lymphoma: Plerixafor + G-CSF Group: 27% had prior therapy once, 52% had prior therapy twice, 21% had prior therapy more than 3 times, 13% had prior radiotherapy;  G-CSF alone Group: 24% had prior therapy once, 58% had prior therapy twice, 18% had prior therapy more than 3 times, 15% had prior radiotherapy. |  |
| Morris | 2020 | NA | Chemotherapy prior to first mobilization: 98.7% of G-CSF + plerixafor，100% of G-CSF alone |  |
| Ray | 2020 | Non-cooperation, bathroom urgency, rigor, blockage of access/return line, etc. (n= 23) | Patients who had had prior radiation therapy, any comorbidities rendering them at high risk for treatment complications or acute infections, were excluded |  |
| Sureda | 2020 | NA | Predicted poor mobilizers were defined as patients who had received prior irradiation to marrow bearing areas or had high exposure to marrow-damaging chemotherapy.  Predicted poor mobilizers: 25% of G-CSF + plerixafor group, 41.2% of G-CSF alone group |  |
| Nahi | 2019 | Received G-CSF but were not assigned to a treatment group due to low CD34+ cell count on day 4 (n = 3) | A potentially poor mobilizer was defined as a person who prior to study entry in the judgment of the investigator may not mobilize HSCs successfully for reasons such as prior lenalidomide administration. |  |
| Matsue | 2018 | Screening failures (n = 5) | Prior surgery therapy: 6.3% of G-CSF + plerixafor group, 18.8% of G-CSF alone group; Prior chemotherapy: 100% of G-CSF + plerixafor group, 100% of G-CSF alone group; Prior radiotherapy: 12.5% of G-CSF + plerixafor group, 12.5% of G-CSF alone group. |  |
| Zhu | 2018 | Adverse events, poor compliance, or other reasons (n = 12) | Previous treatment： Prior surgery therapy: 100% of Plerixafor + G-CSF group, 100% of G-CSF alone group; Prior chemotherapy: 100% of Plerixafor + G-CSF group, 100% of G-CSF alone group; Prior radiotherapy: 100% of Plerixafor + G-CSF group, 14% of G-CSF alone group. |  |
| Haverkos | 2017 | NA | Prior chemotherapy: 2.1% of G-CSF alone group, 3.9% of Plerixafor + G-CSF group; Prior radiotherapy: 31% of G-CSF alone group, 27% of Plerixafor + G-CSF. |  |
| Ri | 2017 | Active infection (n = 1) A reason other than adverse events (n = 2) Splenomegaly and an increase of WBC after the frst plerixafor administration (n = 1) Patient refused for further apheresis after 2 days (n = 2). | All patients received prior anticancer therapy, chemotherapy, surgery therapy, and radiation therapy. |  |
| Sánchez-Ortega | 2015 | NA | G-CSF alone group: 85% had not been mobilized; 15% had been mobilized more than 1 time;  Plerixafor + G-CSF group: 34% had not been mobilized; 66% had been mobilized more than 1 time. |  |
| Maziarz | 2013 | NA | All patients received radiation therapy. |  |
| Micallef | 2013 | NA | All patients received chemotherapy. |  |
| Andreola | 2012 | NA | None of the patients had received any mobilisation therapy before. |  |
| Varmavuo | 2012 | NA | Prior rituximab therapy: Stem cell mobilization with plerixafor (90%); Stem cell mobilization without plerixafor (79%) |  |
| Cooper | 2011 | NA | MM: 18 of more than 4 cycles lenalidomide; 4 of radiotherapy; NHL: 10 of previous stem cell toxic therapy (Includes fludarabine, cisplatin, high-dose cytosine arabinoside, bendamustine, lenalidomide), 4 of radiotherapy; HL: 1 of radiotherapy. |  |
| Horwitz | 2011 | NA | Prior chemotheray: all patinets |  |
| Worel | 2011 | NA | MM: had prior chemotherapy 6 cycles; 13% had prior radiation therapy, and had previous PBSC mobilization attempts once; NHL: had prior chemotherapy 9 cycles, 12% had prior radiation therapy, and had previous PBSC mobilization attempts once; HL: had prior chemotherapy 10 and12 cycles, had no prior radiation therapy, and had previous PBSC mobilization attempts once. |  |
| DiPersio a | 2009 | Intercurrent illness (n = 1) Elective withdrawal (n = 10) Died (n = 14)  Failed mobilization (n = 4)  Lost to follow-up (n = 1) other (n = 15) | Prior chemotherapy: 96.1% of G-CSF alone group, 97.3% of Plerixafor + G-CSF group;  Prior cytotoxic chemotherapy (Cytotoxic chemotherapy included prior treatment with any of the following agents: anthracycline, bortezomide, cyclophosamide, melphalan, and vincristine): 31.2% of G-CSF alone group, 34.5% of Plerixafor + G-CSF group;  Prior radiotherapy: 30.5% of G-CSF alone group, 27% of Plerixafor + G-CSF group. |  |
| DiPersio b | 2009 | Adverse events (n = 4) other (n = 9) Noncompliance (n = 1) Entered rescue procedure (n = 62) Died (n = 28) Failed mobilization (n = 6) Elective withdrawals (n = 4) Lost to follow-up (n = 4) | Prior surgery: 99.3% of Plerixafor +G-CSF group, 100% of G-CSF group; Prior chemotherapy: 96.7% of Plerixafor +G-CSF group, 94.8% of G-CSF group; Prior radiotherapy: 16.7% of Plerixafor +G-CSF group, 19.6% of G-CSF group. |  |
| Stewart | 2009 | NA | Prior chemotherapy: 100% patients with NHL, 100% patients with MM; Prior radiotherapy: 0% patients with NHL, 21.4% patients with MM; cytotoxic chemotherapy: 100% patients with NHL, 94.3% patients with MM. |  |
| Stiff | 2009 | NA | prior cytotoxic chemotherapy: 100% patients with NHL, 61.5% patients with MM; prior radiotherapy: 30.4% patients with NHL, 30.8% patients with MM; first remission: 36.4% patients with NHL, 76.9% patients with MM; extensive radiotherapy: 47.8% patients with NHL, 38.5% patients with MM. |  |
| Cashen | 2008 | NA | Number of prior chemotherapy regimens:  1: 9% of G-CSF + Plerixafor group, 17% of G-CSF alone group; 2: 82% of G-CSF + Plerixafor group, 60% of G-CSF alone group; 3: 9% of G-CSF + Plerixafor group, 20% of G-CSF alone group; more than 4: 0% of G-CSF + Plerixafor group, 2% of G-CSF alone group. Prior radiotherapy: 41% of G-CSF + Plerixafor group, 48% of G-CSF alone group. |  |
| Pusic | 2008 | NA | Prior radiotherapy: 17.4% patients with NHL/HL; 49.6% patients with MM. |  |

G-CSF, granulocyte colony-stimulating factor; HL, Hodgkin’s lymphoma; MM, multiple myeloma; NA, not available; NHL, non-Hodgkin’s lymphoma. PBSC, peripheral blood stem cell.
